# Chest X-ray for predicting mortality and the need for ventilatory support in COVID-19 patients presenting to the emergency department

**DOI:** 10.1007/s00330-020-07270-1

**Published:** 2020-10-08

**Authors:** Maurizio Balbi, Anna Caroli, Andrea Corsi, Gianluca Milanese, Alessandra Surace, Fabiano Di Marco, Luca Novelli, Mario Silva, Ferdinando Luca Lorini, Andrea Duca, Roberto Cosentini, Nicola Sverzellati, Pietro Andrea Bonaffini, Sandro Sironi

**Affiliations:** 1 Department of Radiology, Azienda Socio-Sanitaria Territoriale Papa Giovanni XXIII, Bergamo, Italy; 2 University of Milano-Bicocca, Milan, Italy; 3 Bioengineering Department, Istituto di Ricerche Farmacologiche Mario Negri IRCCS, Bergamo, Italy; 4 Scienze Radiologiche, Department of Medicine and Surgery, University of Parma, Parma, Italy; 5 Respiratory Unit, Azienda Socio-Sanitaria Territoriale Papa Giovanni XXIII, Bergamo, Italy; 6 Department of Health Sciences, University of Milan, Milan, Italy; 7 Department of Anesthesia and Intensive Care, Azienda Socio-Sanitaria Territoriale Papa Giovanni XXIII, Bergamo, Italy; 8 Emergency Department (Emergenza Alta Specializzazione), Azienda Socio-Sanitaria Territoriale Papa Giovanni XXIII, Bergamo, Italy

**Keywords:** Radiography, COVID-19, Severe acute respiratory syndrome coronavirus 2

## Abstract

**Objectives:**

To evaluate the inter-rater agreement of chest X-ray (CXR) findings in coronavirus disease 2019 (COVID-19) and to determine the value of initial CXR along with demographic, clinical, and laboratory data at emergency department (ED) presentation for predicting mortality and the need for ventilatory support.

**Methods:**

A total of 340 COVID-19 patients who underwent CXR in the ED setting (March 1–13, 2020) were retrospectively included. Two reviewers independently assessed CXR abnormalities, including ground-glass opacities (GGOs) and consolidation. Two scoring systems (*Brixia* score and percentage of lung involvement) were applied. Inter-rater agreement was assessed by weighted Cohen’s kappa (*κ*) or intraclass correlation coefficient (ICC). Predictors of death and respiratory support were identified by logistic or Poisson regression.

**Results:**

GGO admixed with consolidation (*n* = 235, 69%) was the most common CXR finding. The inter-rater agreement was almost perfect for type of parenchymal opacity (*κ* = 0.90), *Brixia* score (ICC = 0.91), and percentage of lung involvement (ICC = 0.95). The *Brixia* score (OR: 1.19; 95% CI: 1.06, 1.34; *p* = 0.003), age (OR: 1.16; 95% CI: 1.11, 1.22; *p* < 0.001), PaO_2_/FiO_2_ ratio (OR: 0.99; 95% CI: 0.98, 1; *p* = 0.002), and cardiovascular diseases (OR: 3.21; 95% CI: 1.28, 8.39; *p* = 0.014) predicted death. Percentage of lung involvement (OR: 1.02; 95% CI: 1.01, 1.03; *p* = 0.001) and PaO_2_/FiO_2_ ratio (OR: 0.99; 95% CI: 0.99, 1.00; *p* < 0.001) were significant predictors of the need for ventilatory support.

**Conclusions:**

CXR is a reproducible tool for assessing COVID-19 and integrates with patient history, PaO_2_/FiO_2_ ratio, and SpO_2_ values to early predict mortality and the need for ventilatory support.

**Key Points:**

*• Chest X-ray is a reproducible tool for assessing COVID-19 pneumonia.*

•* The Brixia score and percentage of lung involvement on chest X-ray integrate with patient history, PaO*_*2*_*/FIO*_*2*_ *ratio, and SpO*_*2*_ *values to early predict mortality and the need for ventilatory support in COVID-19 patients presenting to the emergency department.*

**Electronic supplementary material:**

The online version of this article (10.1007/s00330-020-07270-1) contains supplementary material, which is available to authorized users.

## Introduction

Coronavirus disease 2019 (COVID-19) has been declared a pandemic emergency by the World Health Organization on March 11, 2020 [1]. This infectious disease can result in a range of clinical outcomes, from an asymptomatic or mild flu-like illness to severe pneumonia, multiorgan failure, and even death [2]. The diagnosis of COVID-19 is based on the detection of the severe acute respiratory syndrome coronavirus 2 (SARS-CoV-2) by real-time reverse transcriptase–polymerase chain reaction (RT-PCR) testing, most commonly of a nasopharyngeal swab [3]. However, this method has some limitations: it is not universally available, turnaround times can be lengthy, and reported sensitivities vary (30–70%) [4, 5].

In the context of the COVID-19 pandemic, imaging has turned out to be a valuable complementary tool to “rule-in” or “rule out” suspected COVID-19 patients, potentially accelerating the speed of diagnosis compared with RT-PCR dynamics [6]. The choice of whether to use chest radiography (CXR) or computed tomography (CT) as a first-line imaging modality for the assessment of COVID-19 depends on factors that vary considerably among scenarios (e.g., local resources, expertise) [7]. In Europe, as well as in the USA, CXR has been extensively used to triage patients with clinical suspicion of COVID-19 [8, 9]. Even though CXR is less sensitive than CT, especially in the early stage of the disease, it is widely available and relatively inexpensive, can be performed at the bedside, and allows relative rapid cleaning and turn-over between patients, thus minimizing the risk of cross-link infection [10].

The spectrum of chest imaging manifestations of COVID-19 on CXR has been extensively described [11, 12]. However, while the utility of CXR in predicting clinical outcomes has been investigated in the severe acute respiratory syndrome (SARS) coronavirus as well as in a variety of other types of pneumonia [13, 14], very few studies have assessed the prognostic value of CXR in COVID-19 patients [15, 16]. Moreover, data about the reproducibility of CXR findings in COVID-19 still lack.

Therefore, our study aimed (a) to evaluate the inter-rater agreement of initial CXR findings in COVID-19 patients presenting to the emergency department (ED) during the early stage of the pandemic and (b) to determine the value of initial CXR findings combined with demographic, clinical, and laboratory data at ED presentation for predicting mortality and the need for ventilatory support in COVID-19 patients.

## Materials and methods

The Institutional Review Board (Comitato Etico di Bergamo, Italy) approved this retrospective observational study and waived the written informed consent.

### Study population

A total of 359 consecutive patients presenting to the EDs of two affiliated hospitals (Papa Giovanni XXIII and San Giovanni Bianco, Bergamo, Italy) between March 1 and 13, 2020, were considered eligible for inclusion. The inclusion criteria were the following: (a) initial CXR performed in the ED setting and (b) final diagnosis of COVID-19 confirmed by a positive RT-PCR test. The exclusion criteria were (a) unavailable clinical or laboratory data (*n* = 5) and (b) non-diagnostic CXR image quality (*n* = 14). Finally, a total of 340 patients were retrospectively enrolled.

### Data collection

Demographic, clinical, and laboratory data were collected from patients’ medical records. The recorded data included the following: age, sex, medical comorbidities, symptoms, clinical and laboratory data within 24 h of ED presentation (including the oxygen saturation [SpO_2_], fraction of inspired oxygen [FiO_2_], arterial partial pressure of oxygen [PaO_2_], and PaO_2_/FiO_2_ ratio), and mode of respiratory support (oxygen mask, continuous positive airway pressure/noninvasive mechanical ventilation, invasive mechanical ventilation). For patients admitted to the intensive care unit, the highest levels of positive end-expiratory pressure, the use of extracorporeal membrane oxygenation, and prone positioning were also recorded. Patient length of stay was calculated by subtracting the date of ED presentation from the date of discharge or death. Patient survival status, as well as the date of death, was obtained from the Regional Healthcare Information System (SISS, Regione Lombardia, Italy) as of May 12, 2020.

### Imaging acquisition and analysis

Images were acquired using digital radiographic systems (Definium 8000, GE Healthcare; FDR AcSelerate, Fujifilm Corporation) with tube voltages ranging from 120 to 150 kVp and by employing automatic exposure control. The imaging data included CXR images acquired in the posteroanterior and lateral (PA/LAT, *n* = 130) or anteroposterior (AP, *n* = 210) projections. The latter was performed when the patient was too unwell to tolerate standing. Only the AP and PA images were selected and retrospectively evaluated by two reviewers (G.M., a thoracic radiologist with 5 years of experience in a referral center; M.B., a fourth-year radiology resident), blinded to patient history other than COVID-19 positivity. Reviewers independently assessed the presence of lung abnormalities, including ground-glass opacities (GGOs), consolidation, and pulmonary nodules [17]. Distribution of GGOs and consolidation was classified as follows: (a) peripheral (involving mainly the peripheral one-third of the lung), central (involving mainly the central two-thirds of the lung), or neither; (b) unilateral or bilateral; (c) upper zone (above the inferior wall of the aortic arch), middle zone (between the inferior wall of the aortic arch and the right inferior pulmonary vein), lower zone (below the right inferior pulmonary vein), or no zonal predominance. The presence of pleural effusion was assessed. The two reviewers were also asked to grade each CXR using the *Brixia* scoring system, an experimental 18-points severity scoring system designed for the assessment of COVID-19 pneumonia [18]. The *Brixia* score is obtained by dividing each lung into 3 zones (upper, middle, and lower zone, as explained above) and then scoring each zone from 0 to 3 based on types of pulmonary infiltrates detected, as follows: 0, no lung abnormalities; 1, interstitial infiltrates; 2, interstitial and alveolar infiltrates (interstitial predominance); 3, interstitial and alveolar infiltrates (alveolar predominance). The terms “interstitial infiltrate” and “alveolar infiltrate” used in the *Brixia* scoring system were reported in the current study as GGO and consolidation, respectively. CXRs showing only abnormalities other than GGOs and consolidation were scored as 0. Pure consolidation was scored as 3. The number of zones involved was also recorded. In addition, the overall extent of GGOs and consolidation was assessed by visually estimating and then averaging the percentage of involvement within each lung.

### Statistical analysis

Patient data and CXR findings were reported as median and interquartile range (IQR) in case of continuous variables, or numbers and frequency distribution (%) in case of binary or categorical variables.

CXR findings’ inter-rater agreement was assessed both in the whole group and in subgroups with AP and PA radiographs by weighted Cohen’s kappa (categorical variables), or intraclass correlation coefficient (ICC) (quantitative variables, namely the number of lung zones involved, *Brixia* score, and percentage of lung involvement). Moreover, the agreement in *Brixia* score and percentage of lung involvement was visualized by correlation and Bland-Altman plots.

Predictors of death and mode of respiratory support were identified among age, sex, comorbidities, duration of symptoms, SpO_2_, and PaO_2_/FiO_2_ ratio, as well as CXR findings (laterality, type of parenchymal opacity, number of lung zones involved, *Brixia* score, and percentage of lung involvement), by logistic and ordinal logistic regression, respectively. In all cases, univariate analyses were first performed to identify possible predictors. All variables with significant contributions at univariate analysis were included in the multivariate analysis, and main predictors were finally identified by reducing the multivariate model using a stepwise model selection technique. CXR findings refer to the most experienced reviewer, and only patients with no missing data were included in the regression analyses.

Significance of the differences in demographic, clinical, and laboratory data between patients with mild (*Brixia* score < 8) and severe (*Brixia* score ≥ 8) CXR findings was assessed by two-tail independent *t* test (continuous variables) or chi-squared test (binary and categorical variables). Significance of the differences in the demographic, clinical, laboratory, and radiological features between deceased and survived patients was assessed by the Mann-Whitney test (numerical variables) or chi-squared test (binary and categorical variables).

Survival curves and pertinent 95% confidence intervals were computed using the Kaplan-Meier method for the whole patient cohort as well as for patients grouped by individual grouping variables (age, sex, number of comorbidities, PaO_2_/FiO_2_ ratio, CXR findings at ED presentation). The significance of the difference between strata was computed by log-rank test. The distribution by the most invasive respiratory support employed of age, PaO_2_/FiO_2_ ratio, *Brixia* score, percentage of lung involvement, and number of lung zones involved was displayed by boxplots.

In all tests, statistical significance was set at *p* < 0.05. All statistical analyses were performed using R software, version 3.6.3.

## Results

The main demographic, clinical, and laboratory features at ED presentation of the 340 COVID-19 patients included in the study are listed in Table 1. Most patients were male (252/340, 74%). The median age was 68 (IQR = 57–76). Arterial hypertension represented the most common comorbidity (162/340, 48%), followed by cardiovascular diseases (86/340, 25%). The median number of days from symptom onset to ED presentation was 7, with the most common symptoms being fever (296/340, 87%), dyspnea (224/340, 66%), and cough (167/340, 49%). The main blood test alterations were lymphocytopenia (131/190, 69%), increased levels of C-reactive protein (323/333; 97%), lactate dehydrogenase (278/306, 91%), and aspartate transaminase (215/331, 65%). The median PaO_2_/FiO_2_ ratio was 238 (IQR = 143–285).

**Table 1:** Summary of data obtained within 24 h of ED presentation in 340 patients with confirmed COVID-19

Total no.	340
RT-PCR, positive initial results/total no. (%)	313/340 (92%)
Age	
Median [IQR], year	68 [57–76]
Distribution, no./total no. (%)	
18–59 year	101/340 (30%)
60–69 year	86/340 (25%)
70–79 year	96/340 (28%)
≥ 80 year	57/340 (17%)
Gender, F, no./total no. (%)	88/340 (26%)
Smoking history, no./total no. (%)	
Never smoked	102/165 (62%)
Former smoker	54/165 (33%)
Current smoker	9/165 (5%)
Comorbidities, no./total no. (%)	
Any	167/215 (78%)
> 2	57/215 (27%)
Arterial hypertension	162/340 (48%)
Cardiovascular disease**	86/340 (25%)
Obesity***	50/215 (23%)
Diabetes	54/340 (16%)
Dyslipidemia	28/340 (8%)
COPD	22/340 (6%)
Chronic renal failure	12/340 (4%)
Neoplasia (active history)	26/340 (8%)
Rheumatic pathology	18/340 (5%)
Immunodepression	20/340 (6%)
Epilepsy	3/340 (1%)
Cirrhosis	6/340 (2%)
Symptoms, no./total no. (%)	
Fever	296/340 (87%)
Cough	167/340 (49%)
Dyspnea	224/340 (66%)
Pharyngodynia	9/340 (3%)
Asthenia	77/340 (23%)
Anorexia	18/340 (5%)
Myalgia	12/340 (4%)
Diarrhea	19/340 (6%)
Nausea	15/340 (4%)
Vomit	16/340 (5%)
Dizziness	18/340 (5%)
Abdominal pain	6/340 (2%)
Chest pain	12/340 (4%)
Duration of symptoms, no. with data	332
Median [IQR], days	7 [5–10]
Laboratory data	
SpO_2_*, no. with data	277
Median [IQR], %	90 [86–94]
PaO_2_/FiO_2_ ratio, no. with data	258
Median [IQR]	238 [143–285]
Distribution, no./total no.	
< 100, severe ARDS	41/258 (16%)
100–200, moderate ARDS	60/258 (23%)
200–300, mild ARDS	113/258 (44%)
> 300, normal	44/258 (17%)
HB, g/dL	
Median [IQR]	13.8 [12.5–14.9]
< 14, no./total no. (%)	182/338 (54%)
> 17, no./total no. (%)	10/338 (3%)
WBC, /mm^3^	
Median [IQR]	6380 [4865–9412]
< 4000, no./total no. (%)	45/338 (13%)
> 10000, no./total no. (%)	66/338 (20%)
Neutrophils	
Median [IQR] (WBC %)	78 [72–86]
Median [IQR] (/mm^3^)	4999 [3428–7665]
< 2000, no./total no. (%)	16/287 (6%)
> 6700, no./total no. (%)	89/287 (31%)
Lymphocytes	
Median [IQR] (WBC %)	13 [8–18]
Median [IQR] (/mm^3^)	805 [570–1088]
< 1000, no./total no. (%)	131/190 (69%)
Monocytes	
Median [IQR] (WBC %)	6[4–8]
Median [IQR] (/mm^3^)	362 [245–554]
< 250, no./total no. (%)	50/190 (26%)
> 800, no./total no. (%)	16/190 (8%)
Eosinophils	
Median [IQR] (WBC %)	0 [0–0.3]
Median [IQR] (/mm^3^)	0 [0–14.2]
> 500, no./total no. (%)	2/190 (1%)
Basophils	
Median [IQR] (WBC %)	0.2 [0.1–0.3]
Median [IQR] (/mm^3^)	11.9 [8.7–23.7]
> 100, no./total no. (%)	3/190 (2%)
PLT, /mm^3^	
Median [IQR]	177,000 [140,000–226,000]
< 150,000, no./total no. (%)	98/321 (31%)
> 400,000, no./total no. (%)	8/321 (2%)
INR	
Median [IQR]	1.07 [1.02–1.15]
> 1.25, no./total no. (%)	34/279 (12%)
aPTT ratio	
Median [IQR]	1.13 [1.02–1.26]
> 1.25, no./total no. (%)	86/306 (28%)
AST, U/L	
Median [IQR]	52 [37–77]
> 40, no./total no. (%)	215/331 (65%)
ALT, U/L	
Median [IQR]	39 [26–60]
> 40, no./total no. (%)	149/335 (44%)
Creatinine, mg/dL	
Median [IQR]	0.93 [0.77–1.23]
> 1.30, no./total no. (%)	76/337 (23%)
Urea, mg/dL	
Median [IQR]	43 [33–65]
> 50, no./total no. (%)	109/285 (38%)
LDH, U/L	
Median [IQR]	404 [314–551]
≥ 250, no./total no. (%)	278/306 (91%)
CRP, mg/dL	
Median [IQR]	12 [6–18]
≥ 1, no./total no. (%)	323/333 (97%)
Fibrinogen, g/dL	
Median [IQR]	0.63 [0.52–0.73]
< 0.150, no./total no. (%)	0/63 (0%)
> 0.400, no./total no. (%)	59/63 (94%)
Na, mEq/L	
Median [IQR]	138 [136–140]
< 136, no./total no. (%)	78/335 (23%)
> 145, no./total no. (%)	9/335 (3%)
K, mEq/L	
Median [IQR]	3.9 [3.6–4.3]
< 3.5, no./total no. (%)	55/331 (17%)
> 5, no./total no. (%)	19/331 (6%)
Cl, mEq/L	
Median [IQR]	101 [98–104]
< 98, no./total no. (%)	47/243 (19%)
> 107, no./total no. (%)	18/243 (7%)

*SpO_2_ values are reported only in cases with FiO_2_ = 0.21. **Including coronary heart disease, cerebrovascular disease, heart failure, and peripheral vascular disease. ***Defined as BMI ≥ 30. Data are reported as median [IQR] (continuous/numerical variables) or number (%) (binary variables). *ARDS*, acute respiratory distress syndrome; *ED*, emergency department; *COVID-19*, coronavirus disease 2019; *RT-PCR*, real-time reverse transcriptase–polymerase chain reaction; *COPD*, chronic obstructive pulmonary disease; *PaO*_*2*_*/FiO*_*2*_ *ratio*, ratio of partial pressure of oxygen to fraction of inspired oxygen; *HB*, hemoglobin; *WBC*, white blood cells; *PLT*, platelets; *INR*, international normalized ratio; *aPTT ratio*, activated partial thromboplastin time ratio; *AST*, aspartate transaminase; *ALT*, alanine transaminase; *LDH*, lactate dehydrogenase; *CRP*, C-reactive protein; *SpO*_*2*_, oxygen saturation; *FiO*_*2*_, fraction of inspired oxygen; *IQR*, interquartile range

All patients underwent CXR and RT-PCR testing within the first 24 h of ED presentation. Initial RT-PCR tests were performed using nasopharyngeal swabs, according to the protocol established by the World Health Organization [3]. A total of 313 (92%) enrolled patients had a positive initial RT-PCR test result, while 27 had a negative one. The latter were found to have a positive result at a second (*n* = 20) or third (*n* = 5) RT-PCR test from a nasopharyngeal swab, up to a maximum of 8 days after ED referral. Only in 2 cases, the diagnosis of COVID-19 was confirmed by a positive RT-PCR test from bronchoalveolar lavage fluid performed 4 and 14 days after the ED presentation, respectively. All of the 27 patients who tested negative on initial RT-PCR had CXR findings suggestive of pneumonia, while 6 patients were negative for both reviewers on CXR and tested positive on initial RT-PCR. There were no statistically significant differences in CXR findings between patients with first positive nasopharyngeal swab and those who became positive afterward.

**Fig. 1:**
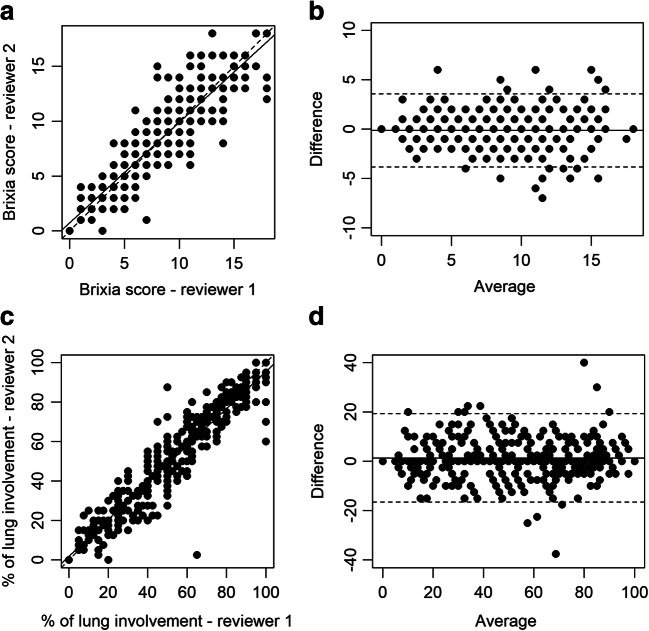
Correlation and agreement between chest X-ray findings obtained by two independent reviewers in 340 patients with confirmed COVID-19. Correlation and Bland-Altman plots show the agreement in *Brixia* score (**a**, **b**) and percentage of lung involvement (**c**, **d**) between the reference reviewer (reviewer 1, a thoracic radiologist with 5 years of experience) and reviewer 2 (a fourth-year radiology resident). In correlation plots, the dashed line denotes the line of perfect concordance, while the solid line denotes the reduced major axis. In Bland-Altman plots, the solid line denotes mean difference, while dashed lines denote mean difference ± 2 standard deviations

**Fig. 2:**
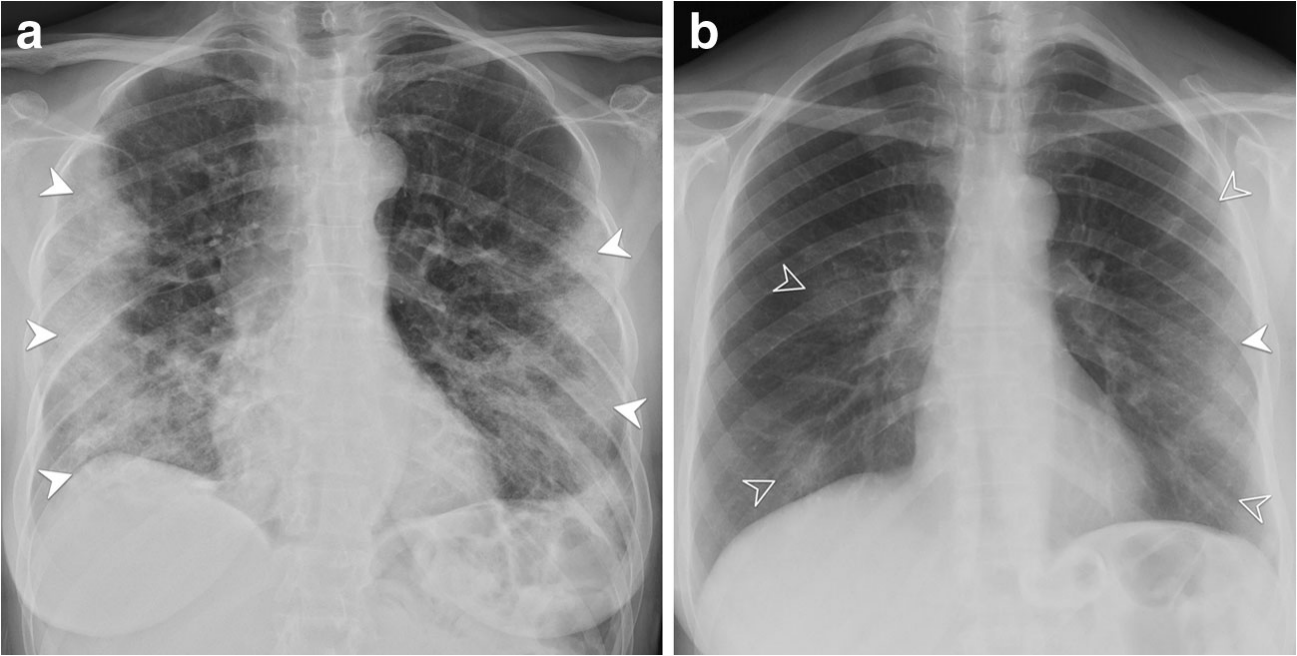
Chest X-ray (CXR) findings at the emergency department presentation in two patients with confirmed COVID-19 and opposite outcomes. **a** CXR shows bilateral, mostly peripheral, ground-glass opacities (GGOs) admixed with consolidation (consolidation-predominant) (arrowheads). Reviewer 1 assigned a *Brixia* score of 14 and a percentage of lung involvement of 60%. Reviewer 2 assigned a *Brixia* score of 15 and a percentage of lung involvement of 50%. This patient had a prolonged stay in the intensive care unit and died 11 days after presenting to the emergency department. **b** CXR shows bilateral GGOs, either pure (empty arrowheads) or admixed with consolidation (GGO-predominant) (solid arrowhead). Reviewer 1 assigned a *Brixia* score of 6 and a percentage of lung involvement of 30%. Reviewer 2 assigned a *Brixia* score of 5 and a percentage of lung involvement of 25%. This patient was discharged from the emergency department after a short-term observation with home care and isolation precautions and was alive at the end of the study period

The inter-rater agreement of CXR findings was almost perfect for the assessment of type of parenchymal opacity (*κ* = 0.90; 95% CI: 0.85, 0.95), *Brixia* score (ICC = 0.91; 95% CI: 0.89, 0.93), and percentage of lung involvement (ICC = 0.95; 95% CI: 0.93, 0.96) [19] (Fig. 1). Notably, AP images showed an overall better inter-rater agreement than PA (Supplementary Material, Table S1). GGO admixed with consolidation was the most common finding (235/340, 69%), followed by GGO (96/340, 28%) (Fig. 2). Parenchymal opacities most frequently showed neither a peripheral nor a central distribution (219 out of 334 with parenchymal opacities, 65%) or were peripherally located (99/334, 30%). Bilateral lung involvement was found in 312 cases (93%) (Table 2). Patients with severe CXR findings more frequently suffered from dyspnea and were more likely to have laboratory abnormalities, including lower SpO_2_ and PaO_2_/FiO_2_ ratio values and raised inflammatory markers, liver enzymes, and creatinine levels (Supplementary Material, Table S2).

**Table 2:** Chest X-ray analysis results obtained by two independent reviewers (reviewer 1, a thoracic radiologist with 5 years of experience; reviewer 2, a fourth-year radiology resident) in 340 patients with confirmed COVID-19

	Reviewer 1	Reviewer 2	Inter-rater agreement	*p*
Normal CXR	6/340(2%)	7/340 (2%)	*κ* = 0.92 [0.77–1.00]	< 0.001
Type of parenchymal opacity			*κ* = 0.90 [0.85–0.95]	< 0.001
GGO	96/340 (28%)	100/340 (29%)		
Consolidation	3/340 (1%)	3/340 (1%)		
GGO and consolidation	235/340 (69%)	230/340 (68%)		
None	6/340 (2%)	7/340 (2%)		
No. lung zones involved	4 [3-6]	4 [4-6]	ICC = 0.86 [0.83–0.88]	< 0.001
≥ 2 lung zones involved	321/340 (94%)	323/340 (95%)	*κ* = 0.71 [0.53–0.88]	< 0.001
Distribution 1			*κ* = 0.78 [0.69–0.86]	< 0.001
Central	16/334 (5%)	8/333 (3%)		
Peripheral	99/334 (30%)	91/333 (27%)		
Neither	219/334 (65%)	234/333 (70%)		
Distribution 2			*κ* = 0.80 [0.72–0.87]	< 0.001
Superior	3/334 (1%)	2/333 (1%)		
Medium	30/334 (9%)	22/333 (6%)		
Inferior	75/334 (22%)	69/333 (21%)		
None	226/334 (68%)	240/333 (72%)		
Distribution 3				
Bilateral	312/334 (93%)	314/333 (94%)	*κ* = 0.71 [0.55–0.87]	< 0.001
Pleural effusion	53/340 (16%)	38/340 (11%)	*κ* = 0.79 [0.69–0.88]	< 0.001
Nodules	3/340 (1%)	1/340 (0.3%)	*κ* = 0.50 [− 0.10 to 1.00]	0.104
Brixia score [18]	7 [4–11]	7 [4–11]	ICC = 0.91 [0.89–0.93]	< 0.001
% of lung involvement	55 [30–76]	54 [28–75]	ICC = 0.95 [0.93–0.96]	< 0.001

The frequency of individual CXR features is reported as number of positive cases or percent distribution. The *Brixia* score and percentage of lung involvement are shown in percent terms and median [IQR], respectively. Inter-rater agreement is shown as weighted Cohen’s kappa (individual CXR features), or intraclass correlation coefficient (no. of lung zones involved, *Brixia* score, and percentage of lung involvement), with pertinent 95% CI. *CXR*, chest X-ray; *GGO*, ground-glass opacity; *IQR*, interquartile range

The main patients’ outcomes are listed in Table 3. Median observation time was 63 days (IQR = 8–67). The two most frequent respiratory supports employed were oxygen mask (144/340, 42%) and continuous positive airway pressure/noninvasive mechanical ventilation (105/340, 31%). Death occurred in 37% of cases (125/340, median age of 76). A total of 58 patients (17%, median age of 60 years) were admitted to ICU, among which 22 died (38%, median age of 66).

**Table 3:** Outcomes of 340 patients with confirmed COVID-19. Follow-up information are reported as of May 12, 2020

Observation time, days	63 [8–67]
Length of hospitalization*, days	7 [4–14]
Respiratory support**	
None	35/340 (10%)
OM	144/340 (42%)
CPAP/NIV	105/340 (31%)
IV	56/340 (17%)
Death, no./total no.	125/340 (37%)
Time to death, days	6 [3–10]
ICU, no./total no.	58/340 (17%)
Age, year	60 [52–66]
Gender, F	14/58 (24%)
PEEP, cm H_2_O ***	16.5 [15–18]
Prone position, no./total no. (%)	26/41 (63%)
ECMO, no./total no. (%)	2/48 (4%)
Deaths, no./total no. (%)	22/58 (38%)

*n = 33 and ***n = 12 data missing due to patients’ transfer to other hospital. **Refers to the most invasive respiratory support employed during observation time. Data are reported as median [IQR] (continuous/numerical variables) or number (%) (binary variables). *OM*, oxygen mask; *CPAP/NIV*, continuous positive airway pressure/noninvasive mechanical ventilation; *IV*, invasive mechanical ventilation; *ICU*, intensive care unit; *PEEP*, positive end-expiratory pressure; *ECMO*, extracorporeal membrane oxygenation; *IQR*, interquartile range

**Fig. 3:**
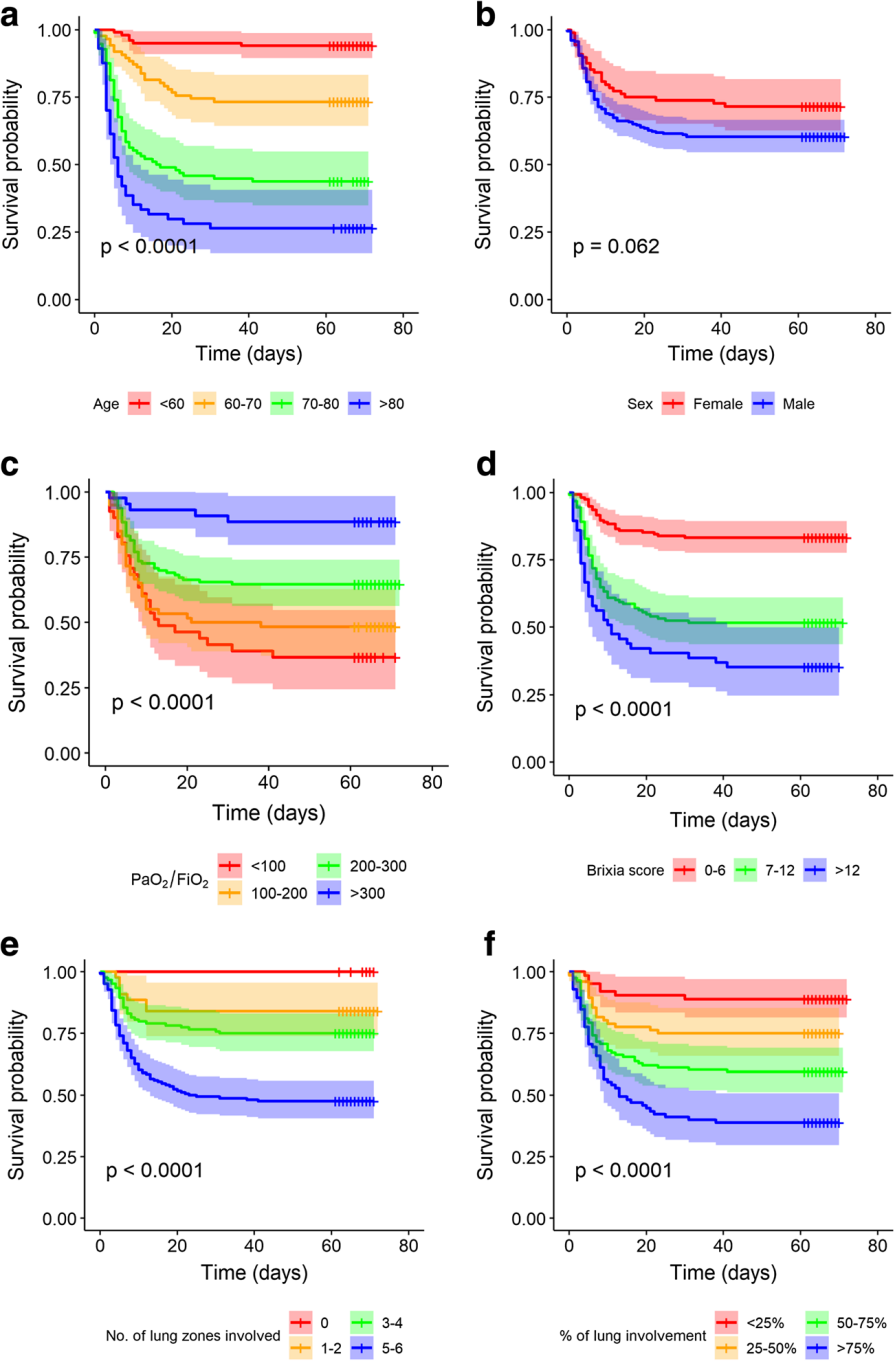
Survival curves related to 340 patients with confirmed COVID-19, grouped by demographic variables (**a**: age, **b**: sex), PaO_2_/FiO_2_ ratio (**c**), and chest X-ray findings at presentation to the emergency department (**d**: *Brixia* score, **e**: number of lung zones involved, **f**: percentage of lung involvement), over a median of 63 days observation time. Shadows denote 95% confidence intervals, while *p* denotes the significance of the difference between strata at log-rank test. PaO_2_/FiO_2_ ratio, ratio of partial pressure of oxygen to fraction of inspired oxygen

Deceased patients were significantly older and had a higher number of comorbidities, significantly lower SpO_2_ and PaO_2_/FiO_2_ ratio values, and more severe CXR findings at ED admission than patients who survived (*p* < 0.001 in all cases; Table 4). Significant differences in survival curves between age classes, PaO_2_/FiO_2_ ratio values, and several CXR findings (*Brixia* score, number of lung zones involved, and percentage of lung involvement) (*p* < 0.001 in all cases) were found (Fig. 3).

**Table 4:** Demographic, clinical, chest X-ray, and laboratory data of 340 patients with confirmed COVID-19 at ED presentation divided in groups based on their clinical outcome: deceased or survived

	Deceased	Survived	*p*
No.	125	215	
Age			
Median [IQR], year	76 [70-82]	61 [54-70]	< 0.001
Distribution, no./total no. (%)			
18–59 year	6/125 (5%)	95/215 (44%)	
60–69 year	23/125 (18%)	63/215 (29%)	
70–79 year	54/125 (43%)	42/215 (20%)	
≥ 80 year	42/125 (34%)	15/215 (7%)	
Gender, F, no./total no. (%)	25/125 (20%)	63/215 (29%)	0.078
Smoking history, no./total no. (%)			0.135
Never smoked	36/61 (59%)	66/104 (63%)	
Former smoker	24/61 (39%)	30/104 (29%)	
Current smoker	1/61 (2%)	8/104 (8%)	
Comorbidities, no./total no. (%)			
Any	73/84 (87%)	94/131 (72%)	0.015
> 2	33/84 (39%)	24/131 (18%)	< 0.001
Arterial hypertension	78/125 (62%)	84/215 (39%)	< 0.001
Cardiovascular disease**	52/125 (42%)	34/215 (16%)	< 0.001
Obesity***	21/84 (25%)	29/131 (22%)	0.749
Diabetes	32/125 (26%)	22/215 (10%)	< 0.001
COPD	11/125 (9%)	11/215 (5%)	0.270
Chronic renal failure	8/125 (6%)	4/215 (2%)	0.060
Neoplasia (active history)	15/125 (12%)	11/215 (5%)	0.037
CXR findings			
Brixia score [18]	10 [7–14]	6 [4–10]	< 0.001
% of lung involvement	70 [50–85]	45 [23–66]	< 0.001
Type of parenchymal opacity			< 0.001
GGO	13/125 (11%)	83/215 (39%)	
Consolidation	3/125 (2%)	0/215 (0%)	
GGO and consolidation	109/125 (87%)	126/215 (58%)	
None	0/125 (0%)	6/215 (3%)	
No. lung zones involved	5 [4–6]	4 [3–6]	< 0.001
Bilateral parenchymal opacities	121/125 (97%)	191/209 (91%)	0.089
Duration of symptoms, no. with data	119	213	0.018
Median [IQR], days	6 [4–9]	7 [5–10]	
Laboratory data at ED presentation			
SpO_2_*, no. with data	90	187	< 0.001
Median [IQR], %	86 [77–89]	92 [89–95]	
PaO_2_/FiO_2_ ratio, no. with data	102	156	
Median [IQR]	179 [97–241]	262 [190–298]	< 0.001
Distribution, no./total no.			
< 100, severe ARDS	26/102 (26%)	15/156 (10%)	
100–200, moderate ARDS	31/102 (30%)	29/156 (18%)	
200–300, mild ARDS	40/102 (39%)	73/156 (47%)	
> 300, normal	5/102 (5%)	39/156 (25%)	
HB, g/dL			
Median [IQR]	13.4 [12.1–14.7]	13.9 [12.6–15.0]	0.033
< 14, no./total no. (%)	73/125 (58%)	109/213 (51%)	
>17, no./total no. (%)	4/125 (3%)	6/213 (3%)	
WBC, /mm^3^			
Median [IQR]	7340 [5050–9820]	6230 [4770–8420]	0.061
< 4000, no./total no. (%)	17/125 (14%)	28/213 (13%)	
> 10000, no./total no. (%)	29/125 (23%)	37/213 (17%)	
Neutrophils			
Median [IQR] (WBC %)	83 [73–88]	77 [71–83]	< 0.001
Median [IQR] (/mm^3^)	5324 [3741–8421]	4627 [3351–6755]	0.058
< 2000, no./total no. (%)	5/111 (4%)	11/176 (6%)	
> 6700, no./total no. (%)	44/111 (40%)	45/176 (26%)	
Lymphocytes			
Median [IQR] (WBC %)	9 [7–14]	15 [10–19]	< 0.001
Median [IQR] (/mm^3^)	662 [546–886]	928 [660–1236]	< 0.001
< 1000, no./total no. (%)	67/82 (82%)	74/108 (59%)	
Monocytes			
Median [IQR] (WBC %)	5 [3–7]	6 [4–8]	0.007
Median [IQR] (/mm^3^)	330 [224–512]	385 [256–557]	0.179
< 250, no./total no. (%)	24/82 (29%)	26/108 (24%)	
> 800, no./total no. (%)	7/82 (9%)	9/108 (8%)	
INR			
Median [IQR]	1.10 [1.05–1.18]	1.05 [1.01–1.11]	< 0.001
> 1.25, no./total no. (%)	19/108 (18%)	15/171 (9%)	
aPTT ratio			
Median [IQR]	1.21 [1.11–1.33]	1.10 [1.00–1.20]	< 0.001
> 1.25, no./total no. (%)	51/118 (43%)	35/188 (19%)	
Creatinine, mg/dL			
Median [IQR]	1.12 [0.81–1.50]	0.90 [0.75–1.04]	< 0.001
> 1.30, no./total no. (%)	44/125 (35%)	32/212 (15%)	
Urea, mg/dL			
Median [IQR]	58 [43–88]	39 [30–48]	< 0.001
> 50, no./total no. (%)	70/110 (64%)	39/175 (22%)	
LDH, U/L			
Median [IQR]	485 [376–633]	372 [294–464]	< 0.001
≥ 250, no./total no. (%)	107/113 (95%)	171/193 (89%)	
CRP, mg/dL			
Median [IQR]	15 [10–22]	10 [4–15]	< 0.001
≥ 1, no./total no. (%)	122/123 (99%)	201/210 (96%)	
K, mEq/L			
Median [IQR]	4.0 [3.7–4.4]	3.9 [3.6–4.2]	0.008
< 3.5, no./total no. (%)	15/122 (12%)	40/209 (19%)	
> 5, no./total no. (%)	13/122 (11%)	6/209 (3%)	

*SpO_2_ reported only in cases with FiO_2_ = 0.21. **Including coronary heart disease, cerebrovascular disease, heart failure, and peripheral vascular disease. ***Defined as BMI ≥ 30. Data are reported as median [IQR] (continuous/numerical variables) or number (%) (binary variables). *P* values are computed Mann-Whitney test (continuous variables), or chi-squared test (binary and categorical variables). *COVID-19*, coronavirus disease of 2019; *COPD*, chronic obstructive pulmonary disease; *PaO*_*2*_*/FiO*_*2*_ *ratio*, ratio of partial pressure of oxygen to fraction of inspired oxygen; *HB*, hemoglobin; *WBC*, white blood cells; *INR*, international normalized ratio; *aPTT*, activated partial thromboplastin time; *LDH*, lactate dehydrogenase; *CRP*, C-reactive protein; *SpO*_*2*_, oxygen saturation; *FiO*_*2*_, fraction of inspired oxygen; *ED*, emergency department; *GGO*, ground-glass opacity; *ARDS*, acute respiratory distress syndrome; *IQR*, interquartile range

**Fig. 4:**
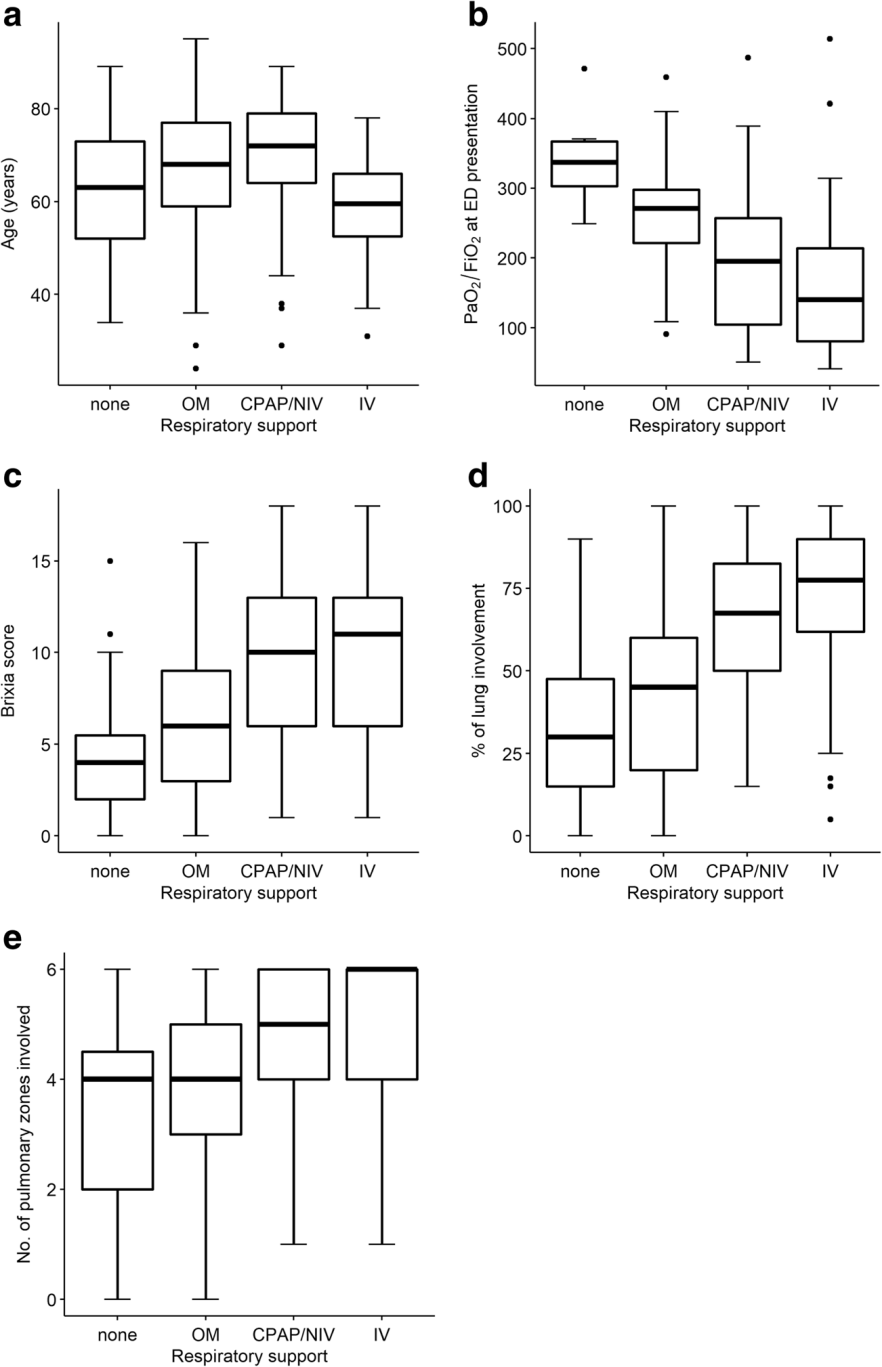
Distribution of age (**a**), PaO_2_/FiO_2_ ratio (**b**), and CXR findings (**c**: Brixia score, **d**: percentage of lung involvement, **e**: number of lung zones involved) at presentation to the emergency department by the most invasive respiratory support employed in 340 patients with confirmed COVID-19. CXR, chest X-ray; ED, emergency department; PaO_2_/FiO_2_ ratio, ratio of partial pressure of oxygen to fraction of inspired oxygen; OM, oxygen mask; CPAP/NIV, continuous positive airway pressure/noninvasive mechanical ventilation; IV, invasive mechanical ventilation

On regression model analysis, the *Brixia* score (OR: 1.19; 95% CI: 1.06, 1.34; *p* = 0.003), age (OR: 1.16; 95% CI: 1.11, 1.22; *p* < 0.001), PaO_2_/FiO_2_ ratio (OR: 0.99; 95% CI: 0.98, 1; *p* = 0.002), and cardiovascular diseases (OR: 3.21; 95% CI: 1.28, 8.39; *p* = 0.014) significantly predicted death. Percentage of lung involvement (OR: 1.02; 95% CI: 1.01, 1.03; *p* = 0.001), SpO_2_ (OR: 0.96; 95% CI: 0.92, 0.99; *p* = 0.008), PaO_2_/FiO_2_ ratio (OR: 0.99; 95% CI: 0.99, 1.00; *p* < 0.001), and rheumatic pathologies (OR: 3.22; 95% CI: 1.05, 9.89; *p* = 0.041) predicted the need for ventilatory support (Table 5). The distribution of age, PaO_2_/FiO_2_ ratio, *Brixia* score, number of lung zones involved, and percentage of lung involvement by respiratory support employed is shown in Fig. 4.

**Table 5:** Demographic, clinical, laboratory, and chest X-ray data that demonstrated predictive value for death and the most invasive respiratory support employed (none, oxygen mask, continuous positive airway pressure/noninvasive ventilation or invasive ventilation) in 340 patients with confirmed COVID-19

	Predictors	Odds ratio	95% CI	*p*
Death	Age, years	1.16	[1.11–1.22]	< 0.001
	SpO_2_, % *	0.96	[0.92–1.01]	0.115
	PaO_2_/FiO_2_ ratio*	0.99	[0.98–1.00]	0.002
	Cardiovascular disease**	3.21	[1.28–8.39]	0.014
	*Brixia* score [18]	1.19	[1.06–1.34]	0.003
Respiratory support	Age, years	0.96	[0.94–0.98]	0.001
	SpO_2_, % *	0.96	[0.92–0.99]	0.008
	PaO_2_/FiO_2_ ratio *	0.99	[0.99–1.00]	< 0.001
	Rheumatic pathology	3.22	[1.05–9.89]	0.041
	% of lung involvement	1.02	[1.01–1.03]	0.001

*Within 24 h of ED presentation. **Including coronary heart disease, cerebrovascular disease, heart failure, and peripheral vascular disease. SpO_2_ reported only in cases with FiO_2_ = 0.21. Odds ratios, 95% CI, and *p* values were computed by logistic (death) and ordinal logistic (respiratory support) regression models, using stepwise model selection technique. Only patients with no missing data (*n* = 210) were included in the models. *ED*, emergency department; *PaO*_*2*_*/FiO*_*2*_ *ratio*, ratio of partial pressure of oxygen to fraction of inspired oxygen; *SpO*_*2*_, oxygen saturation

## Discussion

The ongoing COVID-19 pandemic has highlighted the need for prompt diagnostic and prognostic strategies to optimize patient management, especially when the availability of critical care resources is limited or overwhelmed. In the present study, the vast majority of COVID-19 patients (334/340, 99%) had signs of pneumonia on CXR, even those who tested negative at initial RT-PCR. Presence, distribution, and type of parenchymal opacity (i.e., GGO, consolidation, or both), as well as the *Brixia* score and the percentage of lung involvement, were consistently assessed by two independent reviewers with different levels of expertise. We found that a higher *Brixia* score, increasing age, underlying cardiovascular diseases, and lower PaO_2_/FiO_2_ ratio values were significant predictors of death. The main predictors of the need for ventilatory support were found to be a higher percentage of lung involvement on CXR, the presence of rheumatic pathologies, and lower SpO_2_ and PaO_2_/FiO_2_ ratio values.

A few studies have examined the value of CXR to predict COVID-19 outcomes. In early May 2020, Borghesi et al introduced the *Brixia* score, an experimental CXR scoring system for quantifying lung abnormalities in COVID-19 pneumonia [18]. High *Brixia* score values have been found to predict in-hospital mortality for COVID-19 [15]. Also, Toussie et al found that a lung zone severity score on the initial CXR was associated with the need for intubation in COVID-19 patients aged 21–50 years [16]. No studies have investigated the value of initial CXR to predict mortality in COVID-19 patients so far. In the present study, we first demonstrated that *Brixia* score on initial CXR is predictive of fatal outcome (based on in-hospital and out-of-hospital deaths) in COVID-19 patients. Moreover, in keeping with Toussie et al [16], we found the percentage of lung involvement to be a predictor of the need for ventilatory support. Although the *Brixia* score and the percentage of lung involvement were significant predictors of both mortality and ventilatory support in the univariate analysis, only one of them remained significant in each outcome’s multivariate model because the two scores provide partially overlapping information. The number of lung zones involved and type of parenchymal opacities were also significantly different between survivors and deceased patients, the latter more frequently presenting with a greater degree of lung involvement and consolidation. Remarkably, the overall inter-rater agreement was better for AP images, where the higher disease severity may have led to relatively obvious CXR findings.

Our study population was mainly composed of patients presenting in a relatively advanced stage of the disease, with a median number of days from symptom onset to ED presentation of 7. The proportion of normal CXRs (6/340, 2%) was, therefore, significantly lower than those reported in previous studies where patients presented earlier in the course of their disease [11, 16, 20]. In accordance with reports showing the highest radiological severity of the disease approximately 6–11 days after the onset of symptoms [21, 22], we found CXR signs of advanced pneumonia in a high proportion of patients: GGO admixed with consolidation (235/340, 69%), bilateral parenchymal opacities (312/334, 93%), and a median percentage of lung involvement of 55. Pleural effusion was recorded in a higher percentage of patients (53/340, 16%) than that previously reported [11].

Unlike previous findings [23], we did not find any significant differences between patients with a positive initial RT-PCR result and those who became positive afterward, both presenting with a high prevalence of GGO admixed with consolidation (68% and 81%, respectively). Moreover, all of the 27 patients who tested negative on initial RT-PCR had CXR findings suggestive of pneumonia, thus underlining the potential of CXR as a valuable complementary diagnostic tool in the first-line work-up of suspected COVID-19 patients.

In accordance with Du et al [24], our findings confirm that older age and cardiovascular diseases predict fatal outcome in COVID-19 patients. As expected, a greater number of comorbidities, hypertension, and diabetes were also found to be associated with death. However, in accordance with previous findings [25], these parameters did not remain significant predictors of mortality in multivariate analysis. In line with a recently published larger series, neither smoking nor obesity (defined as BMI ≥ 30) was found to be associated with death [26].

PaO_2_/FiO_2_ ratio was found to be a significant predictor of death and the need for ventilatory support, while SpO_2_ was a significant predictor of the need for ventilatory support only. PaO_2_/FiO_2_ ratio, as a surrogate of hypoxia, was previously found to be a predictor of death in other types of pneumonia [27] and to appear significantly lower in patients deceased of COVID-19 compared with those who survived [28]. In our cohort, most patients presented with a mild respiratory failure (median PaO_2_/FiO_2_ ratio = 238), and PaO_2_/FiO_2_ ratio at ED presentation was significantly reduced in deceased patients compared with that in survivors (179 vs. 262, *p* < 0.001). Our results provide further evidence in support of the PaO_2_/FiO_2_ ratio as a critical parameter to assess disease severity in patients with severe respiratory symptoms due to COVID-19.

The “protective” role of increasing age against the need for ventilatory support found in the present study can be safely considered artifactual. Reasonably, this result has been influenced by the extraordinary distribution of limited healthcare resources, preferentially allocated to patients with a higher possibility of therapeutic success and life expectancy.

The present study has some limitations. First, in such an emergency, the completeness of data recorded was less than optimal. Moreover, our cohort attended the ED after several days from symptom onset and in a relatively advanced disease stage, thus making the generalizability of our results uncertain. Also, such an imbalance between clinical needs and availability of intensive care resources has reasonably led to discrepancies between disease severity or clinical outcomes and mode of respiratory support employed. Lastly, given the very low number of CT scans performed in our institution in this emergency situation and considering that most of them were not acquired at ED presentation but rather later in the disease course, a comparison between CT and CXR findings was not feasible.

In conclusion, CXR is a reproducible tool for assessing COVID-19. Along with patient history, SpO_2_, and PaO_2_/FiO_2_ ratio values, CXR at ED presentation may help to identify patients at risk for death and ventilatory support, thus enabling to optimize clinical management in high-prevalence settings of the disease.

## Electronic supplementary material

ESM 1 (DOCX 41 kb)

## Abbreviations

COVID-19: Coronavirus disease 2019
CXR: Chest radiography
ED: Emergency department
FiO_2_: Fraction of inspired oxygen
GGO: Ground-glass opacity
ICC: Intraclass correlation coefficient
IQR: Interquartile range
PaO_2_: Arterial partial pressure of oxygen
RT-PCR: Real-time reverse transcriptase–polymerase chain reaction
SARS-CoV-2: Severe acute respiratory syndrome coronavirus 2
SpO_2_: Oxygen saturation
