# Supplementary material for: Chest X-ray for predicting mortality and the need for ventilatory support in COVID-19 patients presenting to the emergency department
Source: Eur Radiol. 2020 Oct 8;31(4):1999–2012. doi: 10.1007/s00330-020-07270-1 (PMC7543667; doi:10.1007/s00330-020-07270-1)
Supplement: Supplementary file 1 — (DOCX 41 kb) [file 330_2020_7270_MOESM1_ESM.docx]

| **Table S1** Chest X-ray (CXR) findings in 340 patients with confirmed COVID-19 divided in groups based on CXR projections | | | |  |
| --- | --- | --- | --- | --- |
| **PA projection (n=130)** |  |  |  |  |
|  | **Reviewer 1** | **Reviewer 2** | **Inter-rater reliability** | **p** |
|  |  |  |  |  |
| **Normal CXR** | 5/130(4%) | 6/130 (4%) | K=0.91[0.72-1.00] | <0.001 |
| **Type of parenchymal opacity** |  |  | K=0.91[0.72-1.00] | <0.001 |
| GGO | 54/130 (41%) | 57/130 (44%) |  |  |
| Consolidation | 0/130 (0%) | 1/130 (1%) |  |  |
| GGO and consolidation | 71/130 (55%) | 66/130 (51%) |  |  |
| None | 5/130 (4%) | 6/130 (4%) |  |  |
| **No. lung zones involved** | 4[3-4] | 4[3-5] | ICC=0.82[0.76-0.87] | <0.001 |
| **≥2 lung zones involved** | 119/130 (92%) | 119/130 (92%) | K=0.70[0.48-0.93] | <0.001 |
| **Distribution 1** |  |  | K=0.75[0.62-0.88] | <0.001 |
| Central | 7/125 (6%) | 4/124 (3%) |  |  |
| Peripheral | 47/125 (37%) | 49/124 (40%) |  |  |
| Neither | 71/125 (57%) | 71/124 (57%) |  |  |
| **Distribution 2** |  |  | K=0.75[0.63-0.87] | <0.001 |
| Superior | 1/125 (1%) | 1/124 (1%) |  |  |
| Medium | 17/125 (14%) | 13/124 (10%) |  |  |
| Inferior | 38/125 (30%) | 36/124 (29%) |  |  |
| None | 69/125 (55%) | 74/124 (60%) |  |  |
| **Distribution 3** |  |  |  |  |
| Bilateral | 112/125 (90%) | 112/124 (90%) | K=0.60[0.36-0.84] | <0.001 |
| **Pleural effusion** | 16/130 (12%) | 13/130 (10%) | K=0.88[0.75-1.00] | <0.001 |
| **Nodules** | 1/130 (1%) | 1/130 (1%) | K=1.00[1.00-1.00] | 0.000 |
| **Brixia score** ^[18]^ | 5[3-7] | 5[3-7] | ICC=0.91[0.87–0.93] | <0.001 |
| **% of lung involvement** | 36[20-50] | 33[18-48] | ICC=0.92[0.88–0.94] | <0.001 |
| **AP projection (n=210)** |  |  |  |  |
|  | **Reviewer 1** | **Reviewer 2** | **Inter-rater reliability** | **p** |
|  |  |  |  |  |
| **Normal CXR** | 1/210(0.5%) | 1/210 (0.5%) | K=1.00[1.00-1.00] | <0.001 |
| **Type of parenchymal opacity** |  |  | K=0.94[0.89-0.99] | <0.001 |
| GGO | 42/210 (20%) | 43/210 (20.5%) |  |  |
| Consolidation | 3/210 (1.5%) | 2/210 (1%) |  |  |
| GGO and consolidation | 164/210 (78%) | 164/210 (78%) |  |  |
| None | 1/210 (0.5%) | 1/210 (0.5%) |  |  |
| **No. lung zones involved** | 5[4-6] | 5[4-6] | ICC=0.85[0.81-0.89] | <0.001 |
| **≥2 lung zones involved** | 202/210 (96%) | 204/210 (97%) | K=0.70[0.43-0.98] | <0.001 |
| **Distribution 1** |  |  | K=0.78[0.68-0.89] | <0.001 |
| Central | 9/209 (4%) | 4/209 (2%) |  |  |
| Peripheral | 52/209 (25%) | 42/209 (20%) |  |  |
| Neither | 148/209 (71%) | 163/209 (80%) |  |  |
| **Distribution 2** |  |  | K=0.83[0.73-0.93] | <0.001 |
| Superior | 2/209 (1%) | 1/209 (0.5%) |  |  |
| Medium | 13/209 (6%) | 9/209 (4%) |  |  |
| Inferior | 37/209 (18%) | 33/209 (16%) |  |  |
| None | 157/209 (75%) | 166/209 (79.5%) |  |  |
| **Distribution 3** |  |  |  |  |
| Bilateral | 200/209 (96%) | 202/209 (97%) | K=0.87[0.69-1.00] | <0.001 |
| **Pleural effusion** | 37/210 (18%) | 25/210 (12%) | K=0.74[0.61-0.87] | <0.001 |
| **Nodules** | 2/210 (1%) | 0/210 (0%) | NA | NA |
| **Brixia score** ^[18]^ | 9[6-12] | 10[6-13] | ICC=0.89[0.86–0.92] | <0.001 |
| **% of lung involvement** | 68[46-83] | 68[46-80] | ICC=0.94[0.92–0.95] | <0.001 |

Results from CXR analysis obtained by two independent raters (reviewer 1, a thoracic radiologist; reviewer 2, a fourth-year radiology resident) in 340 confirmed COVID-19 patients divided in groups based on radiographic projections. The frequency of individual CXR features is reported as number of positive cases or percent distribution. The *Brixia* score and percentage of lung involvement are shown in percent terms and median [IQR], respectively. Inter-rater reliability is shown as weighted Cohen’s Kappa (individual CXR features), or intraclass correlation coefficient (no. of lung zones involved, *Brixia* score, and percentage of lung involvement), with pertinent 95% CI. Abbreviations: AP = anteroposterior, CXR = chest X-ray, GGO = ground-glass opacity, IQR = interquartile range, PA = posteroanterior

| **Table S2** Demographic, clinical, and laboratory data at Emergency Department presentation in 340 patients with confirmed COVID-19 divided in groups based on CXR findings | | | |
| --- | --- | --- | --- |
|  | **Mild** | **Severe** | **p** |
| Total no. | 175 | 165 |  |
| RT-PCR, positive initial results/total no. (%) | 163/175 (93%) | 150/165 (91%) | 0.575 |
| CXR projection – no./total no. (%) |  |  | <0.001 |
| AP | 77/210 (37%) | 133/210 (63%) |  |
| PA | 98/130 (75%) | 32/130 (25%) |  |
| Age |  |  |  |
| Median [IQR] – yr | 66[57-74] | 70[57-79] | 0.050 |
| Distribution – no./total no. (%) |  |  |  |
| 18-59 yr | 56/175 (32%) | 45/165 (27%) |  |
| 60-69 yr | 49/175 (28%) | 37/165 (22%) |  |
| 70-79 yr | 47/175 (27%) | 49/165 (30%) |  |
| ≥ 80 yr | 23/175 (13%) | 34/165 (21%) |  |
| Gender, F – no./total no. (%) | 52/175 (30%) | 36/165 (22%) | 0.124 |
| Smoking history – no./total no. (%) |  |  | 0.655 |
| Never smoked | 52/87 (60%) | 50/78 (64%) |  |
| Former smoker | 29/87 (33%) | 25/78 (32%) |  |
| Current smoker | 6/87 (7%) | 3/78 (4%) |  |
| **Comorbidities** – no./total no. (%) |  |  |  |
| Any | 80/103 (78%) | 87/112 (78%) | 1.000 |
| >2 | 23/103 (22%) | 34/112 (30%) | 0.239 |
| Arterial hypertension | 80/175 (46%) | 82/165 (50%) | 0.531 |
| Cardiovascular disease ** | 37/175 (21%) | 49/165 (30%) | 0.091 |
| Obesity *** | 22/103 (21%) | 28/112 (25%) | 0.639 |
| Diabetes | 22/175 (13%) | 32/165 (19%) | 0.116 |
| Dyslipidemia | 16/175 (9%) | 12/165 (7%) | 0.668 |
| COPD | 12/175 (7%) | 10/165 (6%) | 0.938 |
| Chronic renal failure | 2/175 (1%) | 10/165 (6%) | 0.030 |
| Neoplasia (active history) | 10/175 (6%) | 16/165 (10%) | 0.239 |
| Rheumatic pathology | 9/175 (5%) | 9/165 (5%) | 1.000 |
| Immunodepression | 8/175 (5%) | 12/165 (7%) | 0.408 |
| Epilepsy | 2/175 (1%) | 1/165 (1%) | 1.000 |
| Cirrhosis | 3/175 (2%) | 3/165 (2%) | 1.000 |
| **Symptoms** – no./total no. (%) |  |  |  |
| Fever | 151/175 (86%) | 145/165 (88%) | 0.783 |
| Cough | 95/175 (54%) | 72/165 (44%) | 0.064 |
| Dyspnea | 99/175 (56%) | 125/165 (76%) | <0.001 |
| Pharyngodynia | 6/175 (3%) | 3/165 (2%) | 0.558 |
| Asthenia | 44/175 (25%) | 33/165 (20%) | 0.316 |
| Anorexia | 14/175 (8%) | 4/165 (2%) | 0.040 |
| Myalgia | 8/175 (5%) | 4/165 (2%) | 0.436 |
| Diarrhea | 13/175 (7%) | 6/165 (4%) | 0.200 |
| Nausea | 12/175 (7%) | 3/165 (2%) | 0.046 |
| Vomit | 13/175 (7%) | 3/165 (2%) | 0.029 |
| Dizziness | 13/175 (7%) | 5/165 (3%) | 0.117 |
| Abdominal pain | 5/175 (3%) | 1/165 (1%) | 0.245 |
| Chest pain | 8/175 (5%) | 6/165 (2%) | 0.436 |
| Duration of symptoms, no. with data | 173 | 159 |  |
| Median [IQR], days | 7[4-10] | 7[5-10] | 0.652 |
| **Laboratory data** |  |  |  |
| SpO_2_ *, no. | 153 | 124 |  |
| Median [IQR], % | 92[88-95] | 88[80-91] | <0.001 |
| PaO_2_/FiO_2_ ratio, no. | 127 | 131 |  |
| Median [IQR] | 270[219-302] | 185[96-244] | <0.001 |
| Distribution – no./total no. |  |  |  |
| <100 - severe ARDS | 7/127 (6%) | 34/131 (26%) |  |
| 100-200 - moderate ARDS | 19/127 (15%) | 41/131 (31%) |  |
| 200-300 - mild ARDS | 64/127 (50%) | 49/131 (37%) |  |
| >300 – normal | 37/127 (29%) | 7/131 (5%) |  |
| HB, g/dL |  |  |  |
| Median [IQR] | 13.9[12.6-15.0] | 13.6[12.4-14.8] | 0.156 |
| <14 – no./total no. (%) | 90/174 (52%) | 92/164 (56%) |  |
| >17 – no./total no. (%) | 4/174 (3%) | 6/164 (4%) |  |
| WBC, /mm^3^ |  |  |  |
| Median [IQR] | 5905[4482-7932] | 7255[5488-10262] | 0.007 |
| <4000 – no./total no. (%) | 29/174 (17%) | 16/164 (10%) |  |
| >10000 – no./total no. (%) | 24/174 (14%) | 42/164 (26%) |  |
| Neutrophils |  |  |  |
| Median [IQR] (WBC %) | 76[69-83] | 82[74-88] | 0.007 |
| Median [IQR] (/mm^3^) | 4444[3199-6346] | 5442[4047-8492] | 0.006 |
| <2000 – no./total no. (%) | 8/149 (5%) | 8/138 (6%) |  |
| >6700 – no./total no. (%) | 34/149 (23%) | 55/138 (40%) |  |
| Lymphocytes |  |  |  |
| Median [IQR] (WBC %) | 15[9-20] | 10[7-15] | 0.003 |
| Median [IQR] (/mm^3^) | 868[617-1130] | 726[552-1030] | 0.688 |
| <1000 – no./total no. (%) | 62/93 (67%) | 69/97 (71%) |  |
| Monocytes |  |  |  |
| Median [IQR] (WBC %) | 6[4-8] | 5[3-8] | 0.063 |
| Median [IQR] (/mm^3^) | 346[257-517] | 477[214-588] | 0.503 |
| <250 – no./total no. (%) | 21/93 (23%) | 29/97 (30%) |  |
| >800 – no./total no. (%) | 5/93 (5%) | 11/97 (11%) |  |
| Eosinophils |  |  |  |
| Median [IQR] (WBC %) | 0[0-0.2] | 0[0-0.3] | 0.569 |
| Median [IQR] (/mm^3^) | 0[0-10.6] | 0[0-19.6] | 0.165 |
| >500 – no./total no. (%) | 0/93 (0%) | 2/97 (2%) |  |
| Basophils |  |  |  |
| Median [IQR] (WBC %) | 0.2[0.1-0.3] | 0.2[0.1-0.3] | 0.994 |
| Median [IQR] (/mm^3^) | 11.0[8.6-20.4] | 13.0[8.9-27.8] | 0.288 |
| >100 – no./total no. (%) | 2/93 (2%) | 1/93 (1%) |  |
| PLT /mm^3^ |  |  |  |
| Median [IQR] | 171500[137750-222500] | 181000[144000-238000] | 0.112 |
| <150000 – no./total no. (%) | 49/164 (30%) | 49/177 (31%) |  |
| >400000 – no./total no. (%) | 3/164 (2%) | 5/177 (3%) |  |
| INR |  |  |  |
| Median [IQR] | 1.05[1.01-1.12] | 1.09[1.03-1.18] | 0.137 |
| >1.25 – no./total no. (%) | 12/145 (8%) | 22/134 (16%) |  |
| aPTT ratio |  |  |  |
| Median [IQR] | 1.12[1.00-1.23] | 1.15[1.05-1.30] | 0.047 |
| >1.25 – no./total no. (%) | 39/161 (24%) | 47/145 (32%) |  |
| AST, U/L |  |  |  |
| Median [IQR] | 47[33-65] | 56[40-85] | 0.018 |
| >40 – no./total no. (%) | 100/171 (58%) | 115/160 (72%) |  |
| ALT, U/L |  |  |  |
| Median [IQR] | 34[25-52] | 40[28-64] | 0.031 |
| >40 – no./total no. (%) | 70/173 (40%) | 79/162 (49%) |  |
| Creatinine, mg/dL |  |  |  |
| Median [IQR] | 0.90[0.75-1.08] | 1.01[0.80-1.35] | 0.009 |
| >1.30 – no./total no. (%) | 29/173 (17%) | 47/164 (29%) |  |
| Urea, mg/dL |  |  |  |
| Median [IQR] | 39[32-54] | 51[38-73] | 0.057 |
| >50 – no./total no. (%) | 40/147 (27%) | 69/138 (50%) |  |
| LDH, U/L |  |  |  |
| Median [IQR] | 348[286-451] | 463[373-602] | <0.001 |
| ≥250 – no./total no. (%) | 139/160 (87%) | 139/146 (95%) |  |
| CRP, mg/dL |  |  |  |
| Median [IQR] | 9[4-14] | 15[9-20] | <0.001 |
| ≥1 – no./total no. (%) | 163/172 (95%) | 160/161 (99%) |  |
| Fibrinogen, g/dL |  |  |  |
| Median [IQR] | 0.63[0.47-0.72] | 0.65[0.55-0.73] | 0.300 |
| <0.150 – no./total no. (%) | 0/24 (0%) | 0/39 (0%) |  |
| >0.400 – no./total no. (%) | 22/24 (92%) | 37/39 (95%) |  |
| Na, mEq/L |  |  |  |
| Median [IQR] | 138[136-140] | 137[135-141] | 0.647 |
| <136 – no./total no. (%) | 35/173 (20%) | 43/162 (27%) |  |
| >145 – no./total no. (%) | 3/173 (2%) | 6/162 (4%) |  |
| K, mEq/L |  |  |  |
| Median [IQR] | 3.9[3.6-4.2] | 4.0[3.6-4.3] | 0.184 |
| <3.5 – no./total no. (%) | 31/172 (18%) | 24/159 (15%) |  |
| >5 – no./total no. (%) | 10/172 (6%) | 9/159 (6%) |  |
| Cl, mEq/L |  |  |  |
| Median [IQR] | 101[98-103] | 101[98-104] | 0.812 |
| <98 – no./total no. (%) | 27/129 (21%) | 20/114 (18%) |  |
| >107 – no./total no. (%) | 9/129 (7%) | 9/114 (8%) |  |

Summary of data of 340 confirmed COVID-19 patients obtained within 24 hours of ED presentation. Patients with a *Brixia* score value lower than eight were classified as mild, whereas patients with a *Brixia* score value equal to or greater than eight were classified as severe. (*): SpO_2_ values are reported only in cases with FiO_2_ = 0.21. (**): including coronary heart disease, cerebrovascular disease, heart failure, and peripheral vascular disease. (***) defined as BMI ≥ 30. Data are reported as median [IQR] (continuous/numerical variables) or number (%) (binary variables). Abbreviations: ARDS = adult respiratory distress syndrome, CXR = chest X-ray, AP = anteroposterior, PA = posteroanterior, ED = emergency department, COVID-19 = coronavirus disease 2019, RT-PCR = real-time reverse transcriptase–polymerase chain reaction, COPD = chronic obstructive pulmonary disease, PaO_2_/FiO_2_ ratio = ratio of partial pressure of oxygen to fraction of inspired oxygen, HB = hemoglobin, WBC = white blood cells, PLT = platelets, INR = International Normalized Ratio, aPTT ratio = activated partial thromboplastin time ratio, AST = aspartate transaminase, ALT = alanine transaminase, LDH = lactate dehydrogenase, CRP = C-reactive protein, SpO_2_ = oxygen saturation, FiO_2_ = fraction of inspired oxygen, IQR = interquartile range.

P denotes significance of the difference between patients with mild and severe CXR findings assessed by two-tail independent t-test (continuous variables) or chi-squared test (binary and categorical variables)
